# Laparo-Hysterotomy

**Published:** 1882-01

**Authors:** 


### XI.—LAPARO-HYSTEROTOMY.

G. Herz, V.S., writes, in the *Wochenschrift fur Theirheilkunde und Vielzucht*, on the Cæsarian operation performed on a cow. I found the cow suffering from dropsy, with a good appetite and due to calve in about eight days. Advised owner to wait that time, and then kill the cow. After ten days she was taken violently ill, and symptoms showed that death was near. Ascertaining that the calf was alive, I decided to perform the cæsarian operation. Laid the cow on her left side, and to save her from pain had her throat cut. After she had lost blood enough to become benumbed, made an incision thirty-five inches long in the right lower flank, beginning in the rear, cutting upwards, then forward and down, when a large quantity of water, which was in the hollow of the stomach, emptied itself. The womb was opened in the same manner, with the same length of cut, and the fœtus, which laid with its hind quarters towards the natural opening, was taken out. The calf, a male, was of strong build, but was afflicted like the mother, with dropsy of the stomach, which showed itself by the hanging down of the belly. Dropsy in the cow was caused by chronic heart disease.

[This case is similar to the one reported by Dr. Haygard, Case VI., but the necropsy revealed that cardiac lesions were the cause of the ascites. In such cases we would advise the trial of paracentesis abdominalis before resorting to the Cæsarian section. In this case reported by Dr. Herz paracentesis probably would not have saved both cow and calf.—Ed.]

